# Reclassifying Anaphylaxis to Neuromuscular Blocking Agents Based on the Presumed Patho-Mechanism: IgE-Mediated, Pharmacological Adverse Reaction or “Innate Hypersensitivity”?

**DOI:** 10.3390/ijms18061223

**Published:** 2017-06-07

**Authors:** David Spoerl, Haig Nigolian, Christoph Czarnetzki, Thomas Harr

**Affiliations:** 1 Division of Clinical Immunology and Allergy, Department of Medical Specialties, University Hospital and Faculty of Medicine, University of Geneva, rue Gabrielle-Perret-Gentil 4, CH-1205 Geneva, Switzerland; haig.nigolian@hcuge.ch (H.N.); thomas.harr@hcuge.ch (T.H.); 2 Department of Anesthesiology, University Hospital and Faculty of Medicine, University of Geneva, rue Gabrielle-Perret-Gentil 4, CH-1205 Geneva, Switzerland; Christoph.Czarnetzki@hcuge.ch

**Keywords:** drug allergy, perioperative anaphylaxis, pseudo-allergy, adverse drug reaction, mast cell, histamine, Mas-related G-protein coupled receptor member X2

## Abstract

Approximately 60% of perioperative anaphylactic reactions are thought to be immunoglobulin IgE mediated, whereas 40% are thought to be non-IgE mediated hypersensitivity reactions (both considered non-dose-related type B adverse drug reactions). In both cases, symptoms are elicited by mast cell degranulation. Also, pharmacological reactions to drugs (type A, dose-related) may sometimes mimic symptoms triggered by mast cell degranulation. In case of hypotension, bronchospasm, or urticarial rash due to mast cell degranulation, identification of the responsible mechanism is complicated. However, determination of the type of the underlying adverse drug reaction is of paramount interest for the decision of whether the culprit drug may be re-administered. Neuromuscular blocking agents (NMBA) are among the most frequent cause of perioperative anaphylaxis. Recently, it has been shown that NMBA may activate mast cells independently from IgE antibodies via the human Mas-related G-protein-coupled receptor member X2 (MRGPRX2). In light of this new insight into the patho-mechanism of pseudo-allergic adverse drug reactions, in which as drug-receptor interaction results in anaphylaxis like symptoms, we critically reviewed the literature on NMBA-induced perioperative anaphylaxis. We challenge the dogma that NMBA mainly cause IgE-mediated anaphylaxis via an IgE-mediated mechanism, which is based on studies that consider positive skin test to be specific for IgE-mediated hypersensitivity. Finally, we discuss the question whether MRGPRX2 mediated pseudo-allergic reactions should be re-classified as type A adverse reactions.

## 1. Introduction

The term “anaphylaxis” was previously used for IgE-mediated reactions only, whereas the term pseudo-allergic (or anaphylactoid) was used for similar clinical reactions, which occur via a non-IgE-dependent mechanism [1,2]. Both reactions may clinically present with hypotension, bronchospasm, and skin manifestations, typically urticaria [3,4]. The same symptoms might also be seen in cases of non-immune mediated pharmacological adverse drug reactions [5]. As it is not possible to distinguish anaphylactic from pseudo-allergic reactions clinically or by standard allergological investigations, a new definition has been suggested by the European Academy for Allergology and Clinical Immunology (EAACI). Thereby, all immediate-type adverse drug reactions are named anaphylaxis with a further subclassification into allergic or non-allergic [6]. 

The incidence of anaphylactic reactions during general anesthesia has been estimated at 1/4000 to 1/25,000, and about 1/5000 for Neuromuscular blocking agents (NMBA) [7]. The incidence varies according to geographical factors [8], indicating that environmental factors might have an effect on the risk of anaphylaxis during anesthesia. Isolated cutaneous symptoms seem to be more frequent in non-IgE-mediated anaphylaxis, whereas bronchospasm and cardiovascular symptoms are more often seen in IgE-mediated anaphylaxis [2]. However, it has been shown that the anesthetist was able to correctly identify the culprit drug in only one third of all peri-operative reactions [9].

Skin tests have so far been considered to be specific for IgE-mediated hypersensitivity, whereas pseudo-allergic reactions have been considered to yield negative results. However, the term “pseudo-allergy” has been too frequently used to describe any kind of immediate-type allergic-like reaction that is not IgE-mediated [10]. This is particularly the case for non-steroidal anti-inflammatory drug (NSAID) adverse reactions, where skin tests are typically negative due to the lack of mast cell involvement. 

The recent identification of the Mas-related G-protein-coupled receptor member X2 (MRGPRX2), now allows for better classification of different types of non-IgE mediated allergic reactions [11]. According to the EAACI nomenclature, which has been published prior to the description of the MRGPRX2 receptor, these reactions should be classified into non-IgE mediated, allergic hypersensitivity reactions, considering the mast cell as belonging to the immune system [6]. Despite the proposal of the EAACI to abandon the term “pseudo-allergy”, this has been used to describe the reaction resulting from MRGPRX2 activation, and will be used in this review to differentiate this particular pathomechanism from other non-IgE mediated hypersensitivity reactions (Table 1). Especially, NMBA were described to elicit a pseudo-allergic reaction through activation of the MRGPRX2 receptor [11]. Whether this is relevant in human anaphylaxis to NMBA, remains hypothetical. However, this would explain why skin tests with NMBA may be positive in the absence of IgE-mediated hypersensitivity. Therefore, previously published data indicating that most reactions to NMBA are IgE-mediated based on positive skin test should be verified. An underlying pseudo-allergic mechanism would explain the high rate of anaphylactic reactions upon first exposure as well as the high rate of cross-sensitization, mainly demonstrated by positive skin test results to various NMBA [12,13]. 

A better understanding of the underlying mechanism is of clinical relevance for deciding whether the culprit drug may be re-administered or has to be avoided. According to the still generally accepted and widely used classification of adverse drug reactions (ADR) that was developed in the 1970s [14], ADR can be classified into dose-related (“A” for Augmented, type A) or non-dose-related reactions (“B” for Bizarre, type B). The frequency of ADR type A is approximately 80%, whereas type B reactions are rarer [15]. According to this classification, non-IgE mediated hypersensitivity reactions represent type B reactions [16,17]. However, the highly predictable and dose-dependent mast cell degranulation found upon MRGPRX2 activation by NMBA [11] would argue in favor of a re-classification of pseudo-allergic reactions as type A adverse reactions. This would then allow for a more confident re-administration of a specific drug if needed.

## 2. Type A Adverse Drug Reactions Are Often Misinterpreted as Being “Allergic”

Type A ADR are pharmacological adverse reactions, which typically do not involve the immune system, and are therefore predictable. This means that the drug may be re-administered with a lower dose or reduced speed without re-eliciting the same adverse reaction. Examples of type A ADR are diarrhea after antibiotics, or gastric ulcers following prolonged NSAID treatment. Also, toxic reactions are typical type A reactions. The reason why some individuals suffer from type A ADR, whereas others do not, is often unknown. Identifying type A ADR is important, since this will have implications for the future management of the patient. An incorrect labeling as “allergic” may result in withholding optimal treatment for a subsequent illness, which can be deleterious in infectious disease and in anesthetic procedures [15].

## 3. Type B Adverse Drug Reaction Can Be Predictable and Dose Dependent

Type B reactions are ADR that are not predictable. They mainly include hypersensitivity reactions, which are mediated by the immune system and occur in a susceptible subgroup of patients. This susceptibility is classically considered to be due to environmental factors, in particular previous exposure during which the adaptive immune system develops hypersensitivity. However, it has been increasingly noted that some type B reactions that are highly predictable were not due to environmental factors, but to the genetic profile of the patient, linked to particular human leukocyte antigens (HLA). The reason for this is that the antigen presentation to T-cells is dependent on specific HLA haplotypes, for example in abacavir, carbamazepine and allopurinol ADR, which seem to be dose-dependent [18,19].

## 4. Mast Cells as Central Players in IgE-Mediated and Mas-Related G-Protein-Coupled Receptor Member X2-Mediated (MRGPRX2-Mediated) Anaphylactic Reactions

**Figure 1:**
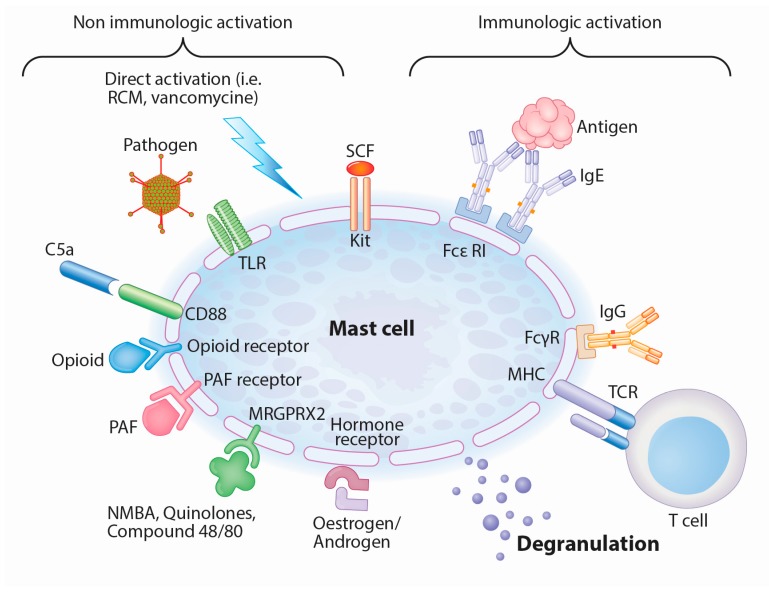
Immunologically and non-immunologically induced mast cell degranulation (adapted from Hannino et al. [20]). Abbreviations: RCM: radiocontrast media, TLR: Toll-like receptor, SCF: Stem cell factor, FcεRI: high affinity IgE receptor, FcγR: IgG receptor, TCR: T-cell receptor, NMBA: neuromuscular blocking agent, PAF: platelet activating factor, MHC: major histocompatibility complex.

Mast cells can release preformed mediators (histamine, serotonin and proteoglycans, mainly heparin), newly formed lipid mediators (thromboxane, prostaglandin D2, leukotriene C4) and cytokines (e.g., tumor necrosis factor alpha, interleukin-4) during anaphylactic reactions. The clinical picture of IgE-mediated hypersensitivity, including hypotension, bronchospasm and urticaria, is mainly caused by these mediators, in particular histamine. However, mast cell degranulation can be also elicited by other mechanisms than IgE crosslinking, as these cells carry a variety of other receptors on their surface that can induce degranulation (Figure 1). Mast cells can be activated by Toll-like receptors (TLR), protease-activated receptors (PARs), opioid receptor, complement (particularly C5a), IgG and under certain circumstances even T-cells, depending on the localization and type of mast cell [20,21]. Whether mast cells are antigen presenting cells, is still matter of debate. Whereas all human mast cell types are activated via the aggregation of high affinity IgE receptors (FcεRI), a subset of mast cells found in the lungs and gut expressing only tryptase do not respond to complement components C3a, C5a and compound 48/80, a polymer used to promote mast cell degranulation [22]. Although TLR-mediated activation of mast cells does not lead to degranulation, but rather to cytokine, chemokine and lipid-mediator production, it is possible that TLR-mediated activation by pathogens may reduce the threshold required for degranulation by other stimuli [23].

## 5. Mast Cells Can Be Stimulated by Various Co-Factors and Mas-related G-Protein-Coupled Receptor Member X2 (MRGPRX2) Receptor Activation

Patients with chronic spontaneous urticaria often suffer from flares caused rather by a non-IgE mediated mechanism than by IgE-mediated allergy [24]. Different eliciting co-factors have been described as possible triggers such as body temperature, infections, hormonal factors, alcohol, or foods. NSAID, opiates, iodinated contrast media, vancomycin, local anesthetics and NMBA represent possible mast cell triggering agents, and should be avoided in patients with chronic urticaria or systemic mastocytosis if possible [1,3,5,25,26,27]. In the recent EAACI position paper on mastocytosis, concerning the use of general anesthetics, the authors state that in the current limited literature, there is conflicting information on both the tolerance of and reactions to different single drugs and drug groups [25]. Other authors suggest to avoid mivacurium and atracurium in particular [28]. 

The combination of different co-factors is thought to have a cumulative effect. This means that a single co-factor alone does not necessarily lead to a clinically apparent mast cell degranulation, but the simultaneous presence of several co-factors have the potential to trigger symptoms mediated by histamine release in these patients [24]. Nevertheless, it is widely accepted that the responsible mechanism does not involve adaptive immunity, and hence does not require sensitization [20]. Similarly, MRGPRX2 induced mast cell degranulation could be considered a co-factor and as such, depend on the presence of other co-factors to be clinically relevant. This mechanism could in turn at least partially explain why only a minority of patients react to NMBA.

## 6. Anaphylactic Reactions upon First Exposure Might Be due to IgE Cross-Sensitization or a Pseudo-Allergic Reaction

Therapeutically used monoclonal antibodies, as well as NMBA, have been described to induce anaphylactic reactions more frequently upon first exposure than subsequent re-exposure. The mechanism of these reactions remains unclear because previous sensitization to the drug is unlikely [29]. For decades, most NMBA were considered to cause non-specific histamine release from mast cells (benzylisoquinolines being more potent histamine releasers than aminosteroidal NMBA), and anesthetists believed that most of these reactions could be prevented by slow injection or pretreatment with antihistamines [30,31]. In recent years, newer studies from allergists, mainly considering positive skin test as a proof for IgE-mediated, type B ADR, indicated that for safety reasons, re-exposure to the culprit drug and to cross-reactive drugs needs to be avoided. 

According to the current concept, a possible explanation for an IgE-mediated reaction upon first exposure could be cross-reactivity among different drugs, as shown in patients reacting to NMBA who have been previously exposed to pholcodine. Pholcodine is a non-prescription antitussive drug that contains a substituted ammonium ion (a moiety where the hydrogen atoms are substituted with other organic groups such as an alkyl group). Epidemiological studies have shown a correlation between the intake of pholcodine and the incidence of NMBA anaphylaxis. This is considered to be due to common substituted ammonium ions (tertiary and/or quaternary ammonium (QA) structures) which are found in a wide variety of chemical structures, including NMBA and pholcodine [8]. The main argument in favor of an IgE-mediated patho-mechanism in perioperative anaphylactic reactions due to NMBA is the reported decrease of the incidence of perioperative anaphylaxis after withdrawal of pholcodine from the market in Norway [32]. In this large cohort the total amount of NMBA exposure was not reported, instead official sales of NMBA in grams were used as indicator of exposure, showing a 12% decrease of NMBA exposure, and in particular a 28% decrease of succinylcholine exposure during the period studied [7]. Though the association between pholcodine exposure and NMBA anaphylaxis seems to be well established, the pathogenic mechanisms connecting these events remain yet to be elucidated [8]. In fact, it has been shown that pholcodine withdrawal was also associated with a decrease of total IgE levels [33], supporting the idea that pholcodine might be a potent “polysensitizer” (polyclonal IgE response) and may elicit NMBA sensitization by another mechanism than by sharing a common epitope. Precisely, mast cell responsiveness might be decreased due to lower circulating total IgE levels, as seen in patients after anti-IgE treatment. Based on this hypothesis, some authors considered the possibility that QA ions might be able to bind directly to immune receptors and stimulate cellular effectors in analogy to the “p-i concept” of drug interaction with majorhistocompatibility complex molecules and T-cell receptors in delayed hypersensitivity reactions [8].

## 7. The Supposed Involvement of the MRGPRX2 Receptor in Adverse Drug Reactions (ADR) to Neuromuscular Blocking Agent (NMBA) Leads to the Hypothesis of an Underlying “Innate Hypersensitivity”

Whereas severe cutaneous ADR associated with particular HLA-types are mainly delayed ADR, the recent identification of the MRGPRX2 receptor has introduced the concept of a genetical, possibly innate, predisposition to develop immediate-type pseudo-allergic ADR. Although environmental influence by epigenetic modification is certainly possible, genetic variants in the *MRGPRX2* gene have already been reported [34,35]. This gene has undergone recent changes during evolution, and eleven haplotypes have been described so far [36]. Three of the four human-specific sequence substitutions are located in extra-cellular domains of the receptor. As extra-cellular receptor domains are usually involved in ligand recognition, the three human-specific amino acid substitutions may markedly modify the interaction between this receptor and its ligands. It is therefore probable that, similarly to several HLA-subtypes associated with drug reactions with eosinophilia and systemic symptoms (DRESS) syndrome or Lyell syndrome, mutations in the *MRGPRX2* gene may be associated with an increased risk for pseudo-allergic ADR.

## 8. Perioperative Anaphylaxis due to NMBA Revised

NMBA are considered to be responsible for the majority of IgE-mediated reactions occurring during general anesthesia, followed by latex, antibiotics, hypnotics and opioids [37,38,39]. Opioids, like morphine, typically trigger non-IgE-mediated reactions [38]. Up to 85% of anaphylactic reactions occur in NMBA-naïve patients [40], and most of the NMBA-allergic patients show a high percentage of cross-reactivity, mostly based on skin tests results [41,42]. However, other authors reported a clinically lack of cross-reactivity between benzylisoquinolines and aminosteroids. Leysen et al. reported that, among 19 allergic patients to rocuronium, 15 were subsequently uneventfully exposed to a benzylisoquinoline [43]. Table 2 shows the most relevant studies indicating the mechanism of anaphylactic reactions to NMBA in chronological order. Most studies consider NMBA to mainly cause IgE-mediated reactions because positive skin tests were considered to prove the presence of IgE [2,43,44,45]. These studies should now be critically reviewed because we know that skin test can be positive in non-IgE mediated hypersensitivity.

### 8.1. Skin Tests to NMBA Have to Be Evaluated with Caution

Skin tests have been so far considered to have a high specificity for IgE-mediated reactions and to be negative in non-IgE mediated, immediate type reactions [2]. In NMBA induced anaphylaxis, this assumption was supported by the observation that most of the patients tolerate NMBA that were negative in skin test [56]. However, cases of a second anaphylactic reaction to a NMBA for which skin tests were negative, have been reported. In one case series, three patients among 192 who reacted to NMBA had a second anaphylactic reaction after re-administration of NMBA which resulted negative in skin test. Another patient had a minor hypersensitivity reaction to an NMBA for which skin tests were negative. Two other patients had additional reactions to NMBA for which they were not tested [42]. This ratio (6/192) is clearly higher than the incidence of anaphylactic reactions in NMBA-naïve patients (i.e., 1/5000), indicating an underlying increased risk of recurrence in these apparently predisposed patients, possibly linked to an underlying “innate” pseudo-allergic mechanism. 

Moreover, patients with immediate type hypersensitivity against antibiotics, confirmed by positive skin test, have an increased risk for positive allergy skin tests for NMBA, independently from their atopic status [57]. This suggests a predisposition to positive skin tests to NMBA in a subgroup of patients without previously known NMBA exposure, possibly due to an underlying pseudo-allergic mechanism. Indeed, skin tests can be positive in patients with pseudo-allergic reactions, in particular when drugs are tested in so-called “irritative concentration” [41]. This seems in agreement with previous data from biopsies of positive skin test to NMBA, in which IgE did not appear to play any role [58]. The fact that most people do not react to non-irritative drug concentrations in skin tests, does not allow to assume that the patients which react have specific IgE against the drug. In a study investigating the cellular and humoral components of NMBA-induced anaphylactic reactions, the correlation between skin test reactivity to rocuronium and IgE to rocuronium was low. In contrast, striking correlation between IgE to rocuronium and skin test reactivity to succinylcholine was found (*p* < 0.001) [52]. This suggests that skin tests with rocuronium might not indicate IgE-mediated allergy, while skin tests to succinylcholine, a drug not acting on the MRGPRX2 receptor but sharing the QA epitope with rocuronium, could be more reliable for true NMBA IgE hypersensitivity. It also shows that IgE to rocuronium might be of lesser clinical relevance, as shown in other studies [43,52]. However, whether skin test with NMBA at correct concentrations may still be of value for IgE-mediated hypersensitivity, remains matter of debate. For rocuronium, a concentration 1/1000 (0.01 mg/mL) has been used in recent studies for intradermal testing and could increase specificity for IgE mediated hypersensitivity [59]. Also, it is not known if MRGPRX2 receptor on human mast cells might have a higher affinity to rocuronium than other NMBA, which could explain the higher rate of reactions to rocuronium than to other NMBA [59].

### 8.2. In Vitro Analysis of NMBA Anaphylaxis Shows Conflicting Results as to the Underlying Mechanism

Tryptase and histamine release are not specific for an IgE-mediated reaction [60,61]. Tryptase increase has been described to be more prominent in IgE-mediated reactions than in non-IgE mediated reactions, but these studies considered positive skin test to be diagnostic for IgE-mediated hypersensitivity [2,55]. Supposing that NMBA (except succinylcholine) might cause pseudo-allergic reactions, these data may be reinterpreted as follows: tryptase increase during the perioperative reaction is associated with an increased likelihood to have positive skin test, irrespective of whether this is mediated by IgE or not. This phenomenon is likely due to an increased propensity of degranulating mast cells during the perioperative reaction and during skin test. 

As to other in vitro diagnostic tools, the value of specific IgE measurements for rocuronium, QA and morphine remains a matter of debate [8,43]. Although specific IgE against morphine appear to have the highest specificity for NMBA hypersensitivity, retrospective data confirm that an isolated positive result for morphine is not a reliable predictor for NMBA allergy [43,62,63]. In particular, specific IgE to morphine is not a good biomarker for sensitization to benzylisoquinolines [64]. In fact, IgE reactivity to tertiary and QA structures has been frequently found in the healthy general population [65]. Moreover, the overall rate of morphine sensitization is quite high, with a prevalence of 10% for patients with non-NMBA allergies and 5% for healthy blood donors in Norway [8]. Although the existence of specific IgE recognizing substituted ammonium ions has been demonstrated based on a radioimmunoassay using a sepharose-alcuronium complex [66], there is no conclusive evidence that conjugation of NMBA or their metabolites to endogenous proteins might form antigenic complexes [29]. Moreover, there is no evidence for any functional role of these antibodies in perioperative anaphylaxis to NMBA in vivo.

Basophil activation test (BAT) appears to be more reliable than measurement of specific IgE [43,44]. If the MRGPRX2 receptor is not expressed on basophils, as presumed by some authors [22], BAT could allow to differentiate true IgE-mediated hypersensitivity to NMBA from pseudo-allergic reaction. More precisely, if BAT would be positive, this would signal true IgE hypersensitivity, while negative BAT would indicate a MRGPRX2 mediated mechanism in a given patient with history of NMBA anaphylaxis. A recent study favored true IgE-mediated anaphylaxis as being responsible for atracurium-induced anaphylaxis because BAT was positive in 5/8 patients with positive skin tests [62]. In another study, BAT was positive in 11/12 patients who suffered from rocuronium-induced anaphylaxis and who had positive skin tests, and in 0/8 patients who tolerated rocuronium and had a negative skin test. This indicates that skin test and BAT results are often coherent and argue against an underlying pseudo-allergic mechanism [67]. However, recent data in literature calls these considerations into question: flow-cytometry studies of basophils recently showed that basophils express MRGPRX2 mainly intracellular [68], and further studies are required to determine this issue.

Atracurium ADR represents an exception among NMBA as the responsible epitope seems to be different than the ammonium ion in IgE-mediated reactions [69,70]. Moreover, taken together it seems that sensitization to atracurium occurs from different routes requiring a prior exposure. Whereas atracurium and mivacurium were already known to cause pseudo-allergic reactions [37], there is now evidence that members of all NMBA families except succinylcholine might cause pseudo-allergic reactions [11]. New data support previous study results showing the histamine releasing potential of several NMBA in healthy subjects, in particular of atracurium, mivacurium, tubocurarine, and to a lesser extent rocuronium [71].

## 9. Discussion

The recent discovery of the MRGPRX2 receptor gave important new insights into the patho-mechanism of pseudo-allergic ADR and questions a few old dogmas, in particular that skin tests are supposed to be specific for IgE-mediated reactions. Therefore, data in the literature reporting ADR based on positive skin tests have to be critically reviewed. Skin testing is not a formal proof for IgE-mediated reactions from the adaptive immune system. A positive skin test can mirror alternative activation pathways of cutaneous mast cell activation. Especially the assumption that anaphylaxis due to NMBA could be mainly IgE-mediated has to be questioned. On one hand the reported decreased incidence of anaphylaxis due to NMBA in Norway after pholcodine withdrawal and the often overlapping results of skin test and BAT, might argue in favor of an IgE-mediated reaction. On the other hand, as discussed in the previous chapters, these studies have several limitations and possible biases, and there are several facts that argue in favor of a pseudo-allergic mechanism: (1) most reactions occur upon first exposure, (2) there is a high rate of cross-sensitization to several NMBA, (3) specific IgE is present in non-allergic individuals without a clear causal link to the anaphylactic reaction, and (4) there is an increased risk of a second anaphylactic reactions to NMBA in skin test-negative patients with previous reaction to another NMBA compared to patients without previous reaction [42]. 

## 10. Conclusions

There is little doubt that IgE against the substituted ammonium ion might be induced by exposure to different chemical substances, i.e., pholcodine, that in turn puts the patient at risk for IgE-mediated anaphylaxis to succinylcholine due to cross-reactivity. However, the recent identification of the MRGPRX2 receptor indicates that other NMBA might induce mainly pseudo-allergic reactions. As mast cell degranulation mediated by MRGPRX2 was found to be dose-dependent and highly predictable [11], we propose that these pseudo-allergic reactions, differently than other non-IgE mediated allergic reactions in which skin test with immediate reading are negative (i.e., T-cell, IgG mediated, eosinophilic), should be reclassified as type A ADRs.

The hypothesis that most NMBA may cause pseudo-allergic rather than IgE-mediated reactions raises several new questions. (1) Supposing that NMBA reactions are due to non-IgE-mediated mast cell degranulation, why do some individuals react more easily than others? (2) Does the expression of MRGPRX2 vary in a single individual over time? Studies addressing the genetic variants and epigenetic modifications of MRGPRX2 are urgently needed to provide answers to these questions and gain more insight in the field of pseudo-allergic reactions. 

## Figures and Tables

**Table 1:** Distinguishing features and proposed classification of different immediate type hypersensitivity reactions.

	Pseudo-Allergic (Mas-Related G-Protein-Coupled Receptor Member X2 (MRGPRX2) Activation)	Non-IgE Mediated, Immunologic Activation (IgG, rarely Described to be Involved in Immediate Type Reaction)	Non-IgE Mediated, Non Immunologic Activation (i.e., Opioid, Complement)	IgE Mediated	Non-Allergic (Immune System Not Primary Involved)
Mast cell involvement	Yes	Yes	Yes	Yes	No
Skin test (immediate reading)	Positive	Negative	Positive	Positive	Negative
Specific IgE	Can be positive without clinical relevance	Can be positive without clinical relevance	Can be positive without clinical relevance	Presumably positive	Can be positive without clinical relevance
Basophil activation test (BAT)	Can be positive without clinical relevance	Presumably negative	For most negative	Presumably positive	Negative
Could explain reaction after first time exposure	Yes	No, except if previous sensitization by cross-reactivity	Yes	No, except if previous sensitization by cross-reactivity	Yes
Dose dependency	Yes	Probably	Yes	Classically no, marginally significant	Yes
Adverse drug reactions (ADR) Classification	New proposal: type A	Type B	Type B	Type B	Type A
Re-administration possible	Theoretically possible, with reduced speed or lower doses. No data available yet	Theoretically not recommended	Theoretically not recommended	Not recommended (consider desensitization protocol)	Yes, with reduced speed or lower doses if not pharmacologically contraindicated

**Table 2:** Major clinical studies with data related to the prevalence and patho-mechanism of adverse drug reactions (ADR) to neuromuscular blocking agents (NMBA) (case series and case reports not considered).

Title	Relevant Data and Remarks
Anaphylactic and anaphylactoid reactions occurring during anesthesia in France in 1999–2000 [2].	Anaphylactic and anaphylactoid reactions were diagnosed in 518 cases (66%) and 271 cases (34%), respectively. The most common causes of anaphylaxis were NMBA (*n* = 306, 58.2%). Anaphylaxis was diagnosed on the basis of clinical history, skin tests, and/or specific immunoglobulin E assay. In case of negative tests, an anaphylactoid reaction was diagnosed.
Anaphylactic and anaphylactoid reactions occurring during anaesthesia in France. Seventh epidemiologic survey (January 2001–December 2002) [46].	Anaphylactic and anaphylactoid reactions were diagnosed in 491 cases (69%) and 221 cases (31%), respectively. The most common causes of anaphylaxis were NMBA (*n* = 271, 55%). Anaphylaxis was diagnosed on the basis of clinical history if skin tests were positive or in case of elevated tryptase values and the presence of specific IgE. In case of negative tests, an anaphylactoid reaction was diagnosed.
Anaphylaxis during Anesthesia in Norway [47].	Eighty-three cases were examined: IgE–mediated anaphylaxis was established in 71.1% of the cases, and NMBA were by far the most frequent culprit drug (93.2%). IgE-mediated anaphylaxis was identified based on a modified categorization grading of causality of the IgE-mediated reactions (investigated by skin prick test, intradermal test, histamine releasing test, specific IgE against morphine and P-aminophenyl phosphoryl choline)
Anaphylaxis during anesthesia: results of a 12-year survey at a French pediatric center [48].	Out of 68 adverse reactions, IgE-mediated anaphylaxis was diagnosed in 51 children: 31 (60.8%) for NMBA, 14 (27%) for latex, seven (14%) for colloids, five (9%) for opioids and six (12%) for hypnotics. IgE-mediated anaphylaxis was diagnosed on the basis of the skin tests results concordant with the patients’ clinical history of adverse reactions and the anesthetic protocol.
Diagnosis of NMBA hypersensitivity reactions using cytofluorimetric analysis of basophils [49].	In 47 NMBA allergic patients, cytofluorimetric analysis of basophils was positive in 17 subjects. The diagnosis of allergy to NMBA was established from a characteristic clinical history (urticaria, bronchospasm and/or anaphylactic shock a few minutes after the start of anesthesia) and the positivity of NMBA skin tests.
Anaphylaxis during general anaesthesia: one-year survey from a British allergy clinic [50].	Out of the 23 patients who presented with anaphylaxis during anesthesia, 15 patients were found to have a positive skin test to at least one NMBA.
Evaluation of a new routine diagnostic test for IgE sensitization to NMBA [51].	In 168 patients exposed to NMBA, quaternary ammonium (QA)-specific IgE was found in 84.2% of skin test-positive reactors. The frequency of QA-specific IgE positivity was significantly higher in skin test-negative reactors (24.6%) than in controls (9.3%), suggesting NMBA sensitivity.
Differentiating the cellular and humoral components of neuromuscular blocking agent-induced anaphylactic reactions in patients undergoing anaesthesia [52].	On the basis of intradermal skin testing and clinical evaluation, allergy to NMBA was considered likely in 48 of 61 patients (79%). Correlation between skin test reactivity to rocuronium and IgE to rocuronium was low. In contrast, striking correlation between IgE to rocuronium and skin test reactivity to succinylcholine was found (*p* < 0.001).
IgE-sensitization to the cough suppressant pholcodine and the effects of its withdrawal from the Norwegian market [32].	Methods used to identify NMBA induced anaphylaxis are not reported. Decrease of perioperative anaphylaxis after pholcodine withdrawal was noted. However, the total amount of NMBA usage was not reported.
Negative predictive value of skin tests to NMBA [53].	55 patients were diagnosed with an allergy to NMBA, confirmed by clinical history, presence of specific IgE and/or positive skin test. 19 of these 55 patients had a second general anesthesia, 13 without NMBA and 6 using an NMBA for which skin tests were negative. None had had a new reaction to the injected NMBA.
Hypersensitivity reactions during anesthesia. Results from the ninth French survey (2005–2007) [45].	An IgE-mediated or non-IgE-mediated reaction was diagnosed in 786 cases (63%) and 467 cases (37%), respectively. The most common causes of anaphylaxis were NMBA (*N* = 373, 47.4%). Allergic or IgE-mediated anaphylaxis was diagnosed on the basis of skin test and/or IgE assay results consistent with the clinical history and the anesthetic protocol.
Perioperative allergic reactions: experience in a Flemish referral centre [54].	Out of 119 patients, a diagnosis of IgE-mediated reaction was established by skin tests and/or specific IgE in 76 cases (63.9%). The most common agents were NMBA (61.8%). The remaining 43 cases (36.1%) were considered as non-IgE-mediated reactions.
Predictive value of allergy tests for NMBA: tackling an unmet need [43].	272 patients with a history of perioperative allergy who had received a NMBA were reported. From the 47 patients who were re-exposed to a NMBA, 19 were initially diagnosed with suspected NMBA allergy, 13 had another IgE-mediated allergy suspected, and in the remainder 15, no IgE-mediated allergy was identified (skin test, specific IgE and BAT were used). Negative skin test and negative BAT assisted the selection of alternative NMBA, which were well tolerated in all cases.
Multi-centre retrospective analysis of anaphylaxis during general anaesthesia in the United Kingdom: aetiology and diagnostic performance of acute serum tryptase [55].	In 161 patients, an IgE-mediated cause was identified in 103 patients (64%); NMBA constituted the leading cause (38%). IgE-mediated reactions were diagnosed based on skin prick test [*n* = 25 (24%)], intradermal test (*n* = 68 (66%)), serum-specific IgE (*n* = 9 (9%)) and challenge tests (*n* = 3 (1%)).
Six years without pholcodine; Norwegians are significantly less IgE-sensitized and clinically more tolerant to NMBA [7].	Five to 10 years after pholcodine withdrawal, very few, if any, individuals were IgE-sensitized to QA ion, and only one case of NMBA-related anaphylaxis per 1–2 years was reported. However, exposure decreased during time of observation and less suxamethonium and more rocuronium were used.

## Abbreviations

NMBA	Neuromuscular blocking agents
NSAID	Non steroidal anti-inflammatory drugs
EAACI	European Academy for Allergology and Clinical Immunology
MRGPRX2	Mas-related G-protein coupled receptor member X2
ADR	Adverse drug reactions
TLR(s)	Toll-like receptors
PAR(s)	Protease-activated receptors
FcεRI	High affinity IgE receptors
QA	Quaternary ammonium
BAT	Basophil activation test
DRESS	Drug reaction with Eosinophilia and systemic symptoms
RCM	Radiocontrast media
SCF	Stem cell factor
FcγR	IgG receptor
TCR	T-cell receptor
PAF	Platelet activating factor
MHC	Major histocompatibility complex
